# Molecular basis of RNA recombination in the 3′UTR of chikungunya virus genome

**DOI:** 10.1093/nar/gkae650

**Published:** 2024-07-25

**Authors:** Eugenia S Bardossy, Sebastiano Volpe, Yasutsugu Suzuki, Fernando Merwaiss, Santiago Faraj, Mónica Montes, Maria-Carla Saleh, Diego E Alvarez, Claudia V Filomatori

**Affiliations:** Escuela de Bio y Nanotecnología, Universidad de San Martín - CONICET, Buenos Aires, Argentina; Institut Pasteur, Université Paris Cité, CNRS UMR3569, Viruses and RNA Interference Unit, 75015 Paris, France; Escuela de Bio y Nanotecnología, Universidad de San Martín - CONICET, Buenos Aires, Argentina; Instituto de Química y Fisicoquímica Biológicas “Prof. Alejandro C. Paladini” (IQUIFIB), Universidad de Buenos Aires - CONICET, Buenos Aires, Argentina; Institut Pasteur, Université Paris Cité, CNRS UMR3569, Viruses and RNA Interference Unit, 75015 Paris, France; Center for Marine Environmental Studies (CMES), Ehime University, Matsuyama, Japan; Institut Pasteur, Université Paris Cité, CNRS UMR3569, Viruses and RNA Interference Unit, 75015 Paris, France; Instituto de Biología Molecular y Celular de Plantas (CSIC-Universitat Politècnica de València), 46022 Valencia, Spain; Instituto de Química y Fisicoquímica Biológicas “Prof. Alejandro C. Paladini” (IQUIFIB), Universidad de Buenos Aires - CONICET, Buenos Aires, Argentina; Instituto de Química y Fisicoquímica Biológicas “Prof. Alejandro C. Paladini” (IQUIFIB), Universidad de Buenos Aires - CONICET, Buenos Aires, Argentina; Institut Pasteur, Université Paris Cité, CNRS UMR3569, Viruses and RNA Interference Unit, 75015 Paris, France; Escuela de Bio y Nanotecnología, Universidad de San Martín - CONICET, Buenos Aires, Argentina; Escuela de Bio y Nanotecnología, Universidad de San Martín - CONICET, Buenos Aires, Argentina; Instituto de Química y Fisicoquímica Biológicas “Prof. Alejandro C. Paladini” (IQUIFIB), Universidad de Buenos Aires - CONICET, Buenos Aires, Argentina

## Abstract

Chikungunya virus (CHIKV) is a rapidly spreading re-emergent virus transmitted from mosquitoes to humans. The emergence of epidemic variants has been associated with changes in the viral genome, such as the duplication of repeated sequences in the 3′ untranslated region (UTR). Indeed, blocks of repeated sequences seemingly favor RNA recombination, providing the virus with a unique ability to continuously change the 3′UTR architecture during host switching. In this work, we provide experimental data on the molecular mechanism of RNA recombination and describe specific sequence and structural elements in the viral 3′UTR that favor template switching of the viral RNA-dependent RNA polymerase on the 3′UTR. Furthermore, we found that a 3′UTR deletion mutant that exhibits markedly delayed replication in mosquito cells and impaired transmission *in vivo*, recombines in reference laboratory strains of mosquitoes. Altogether, our data provide novel experimental evidence indicating that RNA recombination can act as a nucleic acid repair mechanism to add repeated sequences that are associated to high viral fitness in mosquito during chikungunya virus replication.

## Graphical Abstract

**Graphical Abstract:**
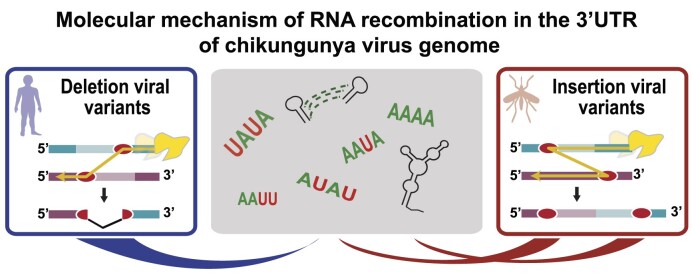

## Introduction

RNA viruses exist in genetically diverse populations. Viral diversity results from both incorporation of point mutations and rearrangement of existing genomes by RNA recombination (1–3). Experimental studies, together with analyses of the constantly growing viral sequence database, have revealed that RNA recombination occurs in many families of RNA viruses, including human, plant, animal, bacterial, insect, and fungal viruses (4). Although recombination is a pervasive process, it has a double edge. On the one hand, recombination is beneficial for the virus, as it facilitates adaptation to unfavorable environments by creating novel genetic combinations. In this regard, it is well documented that RNA recombination plays a key role in the emergence of new virus species and strains (5,6). It might lead to the expansion of host range and cell tropism (7–9), the generation of virulent variants (10–13), the viral escape from vaccine protection (14) and the evasion of host defense mechanisms (15). In addition, RNA recombination functions as a repair mechanism, essential for counteracting mutations incompatible with viral viability and maintaining the integrity of viral genomes (16). On the other hand, RNA recombination can have adverse consequences for the virus, as it distorts part of favorable genomic regions generating deleterious combinations (17,18). Generally, recombination rate is optimized to maintain a balance between beneficial and detrimental effects, thus increasing the adaptability of the virus.

Copy choice is the most widely accepted mechanism of RNA recombination. This process involves the dissociation of the RNA-dependent RNA polymerase (RdRp) and the nascent strand from a donor RNA template, and reassociation on a homologous acceptor RNA molecule. Template switching events are thought to require significant sequence similarity between donor and acceptor RNAs and are therefore commonly referred to as recombination by similarity. For many viruses, the junction points are not randomly distributed. Instead, there are defined hotspot regions that appear to favor the recombination process (19–21). In some cases, the recombination breakpoints are located in the matching homologous sequences of both donor and acceptor templates, so that the product retains the information of the parental RNA. In other cases, the RdRp switches to a different position on the acceptor RNA template, resulting in a chimeric RNA molecule that contains either the duplication or the deletion of the RNA sequence between breakpoints (22). Usually but not always, this type of template switching mechanism generates aberrant or defective viral genomes (23–25).

Chikungunya virus (CHIKV) is a re-emergent virus transmitted to humans by infected mosquitoes (26–28). It is responsible for disease outbreaks in Africa and Asia, and more recently in Europe and the Americas (29). The spread of CHIKV to the Americas has resulted in millions of infections since virus circulation was first detected on the Caribbean Island of Saint Martin in October 2013. Re-emergent CHIKV lineages have fixed mutations in coding sequences and large variations in their 3′UTR (30–32). In particular, the 3′UTR of CHIKV contains short sequence repeats, named direct repeats (DRs), which vary in copy number among viral lineages (33). Notably, genome sequencing of re-emergent viral strains suggests that the natural evolution of CHIKV is linked to the net gain of DR copies (31,34).

Prompted by the notion that repeated sequences could favor RNA recombination, we have recently investigated the 3′UTR composition of CHIKV populations adapted to cultured cells (35). We found that genomic recombination occurs with high frequency at the end of the viral genome, generating a spectrum of viable and non-viable variants with different number of DR copies in their 3′UTR.

Arboviruses, such as CHIKV, have evolved strategies to closely interact with both mammalian and arthropod hosts to perpetuate in nature. These viruses often contain plastic genomes that continuously change during host switching, providing adaptability (36). Importantly, the CHIKV 3′UTR is under opposite selective pressures in mammalian and mosquito hosts (31,34,37). Whereas DRs are dispensable in mammalian cells, they enhance viral replication in mosquito cells (35). Consequently, recombinant viral variants lacking DR copies are widely generated and positively selected in mammalian cells, but cleared from the viral population when the virus replicates in mosquitoes. Based on these results, we have proposed a model to explain the function of the 3′UTR during host switching, in which genomic recombination acts together with natural selection to shape the composition of the viral population, enabling CHIKV to efficiently cross species barriers (35).

Recently, we have gained insight into the relevance of DR copies in mosquitoes. We found that an engineered virus from the Caribbean strain that carried a deletion of DR copies had an impaired ability to cross anatomical barriers in *Aedes* mosquitoes, displaying a delayed release into the mosquito saliva as also reflected by a longer external incubation period compared to the wild type (WT) virus (38). Although the evidence to date suggests the 3′UTR is an important determinant of viral fitness and epidemic potential, the forces that drive evolution of the viral 3′UTR are yet to be determined. What are the molecular signals that promote RNA recombination in the CHIKV 3′UTR? How RNA recombination acts *in vivo* to improve the fitness of CHIKV variants with impaired transmission?

Here, we investigated the interplay between DR copies and RNA recombination in the CHIKV 3′UTR, both *in vitro* and *in vivo*. Infecting mammalian cells with CHIKVs bearing 3′UTR of different lengths, we found a positive correlation between the number of DR copies in the parental strain and the diversity of the CHIKV populations. The analysis of the adapted viral variants revealed that the CHIKV 3′UTR is enriched in RNA elements that favor template switching of RdRp. This process results in the emergence of 3′UTR variants that are positively or negatively selected depending on host-specific demands for viral replication. Importantly, we found that RNA recombination occurs *in vivo* in mosquitoes experimentally infected with a virus with a minimal 3′UTR. Functional studies showed that these emergent variants that acquired DR copies had a clear advantage to replicate in mosquito cells over the mutant virus, underlining the relevance of RNA recombination for host adaptation.

## Materials and methods

### Recombinant CHIKVs

The Δ(1 + 2)a, Δ(1 + 2)ab, Δ(1 + 2)abb’, Mut SLYa and Mut SLYb constructs were generated by overlapping PCR as previously described (35), using a CHIKV-Cbn plasmid containing a unique *Sac*I restriction site downstream of the stop codon of the viral structural proteins (Supplementary Table S1). Rec SLYb was constructed by restriction-free cloning (39) using Mut SLYb as template. This approach involved: (i) a first PCR to amplify a megaprimer, (ii) a second PCR using the megaprimer and Mut SLYb plasmid as a template, and (iii) *Dpn*I digestion and transformation of the second PCR product.

To facilitate the construction of Mut (1 + 2), a unique *BamH*I restriction site was introduced downstream of the DR(1 + 2) copies by restriction-free cloning. Then, a 60% GC *Sac*I-*BamH*I fragment was amplified and used to replace the *Sac*I-*BamH*I fragment of CHIKV-Cbn, as described in Supplementary Table S1.

Recombinant DNA constructs were linearized by digestion with *Not*I and used as templates for transcription with SP6 polymerase in the presence of m^7^G(5′)ppp(5′)G cap structure using the mMessage Machine transcription kit (Thermo Fisher). *In vitro* transcribed RNAs were spectroscopically quantified, and their integrity was verified by electrophoresis on agarose gels.

### Cells and viral transfections

Mammalian BHK-21 cells (*Mesocricetus auratus* hamster kidney, ATCC, CCL-10) were grown at 28°C or 37°C in MEM alpha medium (Gibco) in 5% CO_2_ atmosphere supplemented with 10% of fetal bovine serum (FBS, Gibco) and 1% penicillin–streptomycin (Gibco). Huh-7 cells (Human hepatocyte cell line, ATCC, CVCL_0336) were cultured at 28°C or 37°C in D-MEM high-glucose medium supplemented with 10% of FBS and penicillin-streptomycin. Mosquito C6/36 cells (*Aedes albopictus*, ATCC, CRL-1660) were grown at 28°C in Leibovitz L-15 medium (Gibco) supplemented with 10% of FBS, 10% of tryptose phosphate (Britania), 1% penicillin–streptomycin and amphotericin B (Gibco).

For RNA transfections, cell lines were grown to 60–70% confluence and transfected in 24-well plates using Lipofectamine 2000 (Invitrogen), according to the manufacturer's instructions. Viral stocks were obtained by transfecting 500 ng of *in vitro* transcribed viral RNA and harvested from the cell culture supernatant at different times after transfection.

### Plaque assay

Viruses were quantified from culture supernatants to determine viral yield. 10^5^ Vero cells (*Cercopithecus aethiops* kidney, ATCC, CCL-81) were seeded per well in 24-well plates and allowed to adhere overnight. Viral stocks were serially diluted; 0.1 ml of the diluted viruses was added to the cells and incubated for one hour at 37°C. Then, 1 ml of overlay (1× D-MEM medium, supplemented with 2% of FBS, 1% penicillin-streptomycin and 0.4% of methylcellulose (Sigma)) was added to each well. Cells were fixed 3 days post-infection with 10% formaldehyde and stained with crystal violet (Sigma).

### Experimental host adaptation

*In vitro* transcribed RNAs for WT, Δ(1 + 2)a, Δ(1 + 2)ab, Δ(1 + 2)abb’, Mut SLYa, and Mut SLYb, Rec SLYb and Mut (1 + 2) were transfected into BHK-21 cells. Viruses were harvested after two days, and a second infection was performed in the same cell line at a multiplicity of infection (MOI) = 0.5. The viral RNAs were then Trizol-extracted (Invitrogen) from the culture supernatants and used for RT-PCR reactions with primer 115 (5′-TTTTTTTTTTTTTTTTTTTGAAATAT-3′, complementary to the poly(A) tail plus the last 7 nucleotides of the CHIKV genome). PCR reactions were performed using reverse oligonucleotide 115 and forward oligonucleotide 116 (5′-CTAATCGTGGTGCTATGC-3′, complementary to the last portion of the coding region of the viral genome). The products were ligated into the pCR2.1-TOPO vector (Invitrogen) and used to transform XL1-Blue bacteria. Two independent experiments were performed. In each experiment, 20 clones were analyzed for WT, Δ(1 + 2)a, Δ(1 + 2)ab, Δ(1 + 2)abb’, Mut SLYa, Mut SLYb, Rec SLYb and Mut (1 + 2) viruses. The length of the individual viral 3′UTRs was estimated by resolving the product of the 115–116 PCR amplification on 1.5% agarose gels. Individual plasmid clones corresponding to the recombinant viruses were sequenced by the Sanger method.

To introduce the 3′UTR of selected recombinant variants into the infectious clone, we performed PCR reactions using cloned 3′UTR as template and plasmids were obtained as described in Supplementary Table S1.

### Mosquito rearing

Laboratory colonies of *Aedes albopictus* mosquitoes (19th generation; originally collected in Phu Hoa, Binh Duong Province, Vietnam) were used. Insectary conditions for mosquito maintenance were 28°C, 70% relative humidity, and a 12-h light and 12-h dark cycle. Adults were maintained with permanent access to a 10% sucrose solution. Adult females were offered commercial rabbit blood (BCL, Boisset-Saint-Priest, France) twice a week through a membrane feeding system (Hemotek Ltd.).

### Experimental infections of mosquitoes

Infection assays were conducted in a biosafety level 3 (BSL-3) laboratory using 7- to 10-day-old females starved 24 h prior to infection. Infectious blood meal was offered to mosquitoes for 30 min through a membrane feeding system (Hemotek Ltd) set at 37°C with a piece of desalted pig intestine as the membrane. The blood meal consisted of washed human erythrocytes resuspended in phosphate buffered saline mixed 2:1 with a pre-diluted viral stock supplemented with 10 mM ATP (Sigma-Aldrich). The viral stock was pre-diluted in Leibovitz L-15 medium containing 0.1% sodium bicarbonate (Gibco) to achieve an infectious titer ranging from 1 × 10^6^ to 1 × 10^7^ focus-forming units. After blood meal, fully engorged females were selected and incubated at 28°C with 70% relative humidity and under a 12-h-light/12-h-dark cycle, with permanent access to 10% sucrose. At different times post-infection, mosquitoes were homogenized in microtubes containing steel beads (5 mm diameter) and 300 μl of DMEM supplemented with 2% FBS using a TissueLyser II instrument (Qiagen) at 30 shakes/s for 2 min. Homogenates were clarified by centrifugation and stored at 80°C until processing. To analyze the CHIKV 3′UTR in viral populations, the TRIzol-extracted RNAs were used for reverse transcription reactions with reverse oligonucleotide 115 and then for PCR reactions with oligonucleotides 115 and 116. The length of the individual viral 3′UTRs was estimated by resolving the product of 115–116 PCR amplification on 1.5% agarose gels. Individual plasmid clones corresponding to the recombinant viruses were sequenced by the Sanger method.

### Human blood and ethics statement

Human blood used to feed mosquitoes was obtained from healthy volunteer donors. Donor recruitment was organized by local investigator assessment using medical history, laboratory results and clinical examinations. Biological samples were supplied through participation of healthy volunteers at the ICAReB biobanking platform (BB-0033–00062/ICAReB platform/Institut Pasteur, Paris/BBMRI AO203/[BIORESOURCE]) of the Institut Pasteur to the CoSImmGen and Diagmicoll protocols which have been approved by the French Ethical Committee (CPP) Ile-de-France I. The Diagmicoll protocol was declared to the French Research Ministry under the reference: DC 2008-68 COL 1.

### Sequence alignments, breakpoints identification and structural analysis

The CHIKV 3′UTR sequences were aligned using the ClustalW2 program. The RNA secondary structure was predicted using RNAalifold software. Based on the 3′UTR alignments, the breakpoints for RNA recombination were identified. The SHAPE constrained thermodynamic models were done with RNAstructure program (version 6.4, Mathews lab) considering the consensus 3′UTR sequences and the SHAPE reactivity values obtained by Madden *et al.* (40). Plots of AU content as a function of the nucleotide position in the CHIKV genome, calculated as (A + U)/(A + U + G + C) × 100% using the Biologicscorp program. Internal duplications were identified using Vector Builder program and represented using sequence dot plots.

### Immunofluorescence assays

C6/36 cells were seeded in a 24-well plate with a 1 cm^2^ coverslip inside and transfected with WT RNA, Δ(1 + 2)abb' or Rec9. Immunofluorescence (IF) assays were performed at different times after transfection. To detect viral antigens, cells were fixed in methanol and stained with a 1:1000 dilution of the mouse anti-CHIKV monoclonal antibody CHK-152 (41) in PBS. Goat anti-mouse alexa Fluor 488 (Molecular Probes) was used as secondary antibody at a 1:1000 dilution.

### Growth curves

Subconfluent C6/36 cells in a six-well plate were infected with WT, Δ(1 + 2)abb’ or Rec9 viruses using an MOI = 0.1. One hour post-infection, cells were washed 5 times with PBS (Gibco) and 2 ml of growth medium was added. Cell supernatants were collected at different time points and frozen at –70°C. To quantify viral titers, supernatants were serially diluted and plaque assays were performed on Vero cells.

### *In vitro* competition experiments

The 3′UTRs of selected recombinant viruses were introduced into the parental infectious clone. Then, the parental and recombinant RNAs were obtained by *in vitro* transcription, quantified, and mixed pairwise at defined ratios. A total amount of 3 μg of transcripts per well was transfected into cultured cells in two independent experiments. Viruses were harvested from the supernatants and used to reinfect fresh cells. After one and two passages, RNA was extracted, used as a template for RT-PCR reactions and ligated into the pCR2.1-TOPO vector. The relative abundance of each virus in the population was then calculated.

## Results

### *In vitro* evolution in mammalian cells of the wild type and mutant viruses carrying 3′UTR deletions

**Figure 1.**
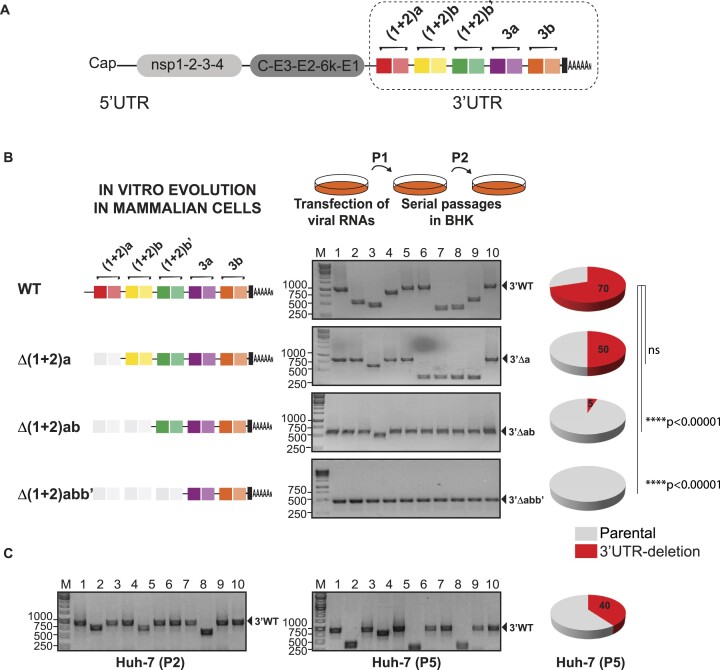
Composition of CHIKV populations grown in mammalian cells. (**A**) Schematic representation of the CHIKV genome. Direct Repeats (DRs) in the 3′UTR are represented with rectangular colored blocks. (**B**) Left, schematic representation of the 3′UTR of WT and the Δ(1 + 2)a, Δ(1 + 2)ab and Δ(1 + 2)abb’ mutant viruses. Two independent *in vitro* evolution experiments were performed for each virus and 20 individual clones were analyzed for each condition. Middle, representative agarose gels for PCR amplification of the 3′UTRs of individual clones recovered from the WT and mutant viral populations passaged twice in BHK cells. The sizes of DNA bands in the ladder (base pairs) and the parental full-length and mutant 3′UTRs are shown for reference. Right, pie charts for the frequencies of parental 3′UTR (gray) and emergent 3′UTR-deletion variants (red) in viral populations. Statistics were performed using Fisher's exact test on cumulative data analyzing each condition vs. the WT (Key: ns, not significant). (**C**) Analysis of the 3′UTR in CHIKV populations after two and five passages of the WT virus in human Huh-7 cells. For each condition, two independent *in vitro* evolution experiments were performed, and 20 individual clones were analyzed.

To investigate the role of DRs in host-specific adaptation of the CHIKV 3′UTR, we used the Asian-derived isolate that arrived to the Americas in 2013 and contains long 3′UTRs with three copies of DR(1 + 2) and two copies of DR3 (Figure 1A). We performed *in vitro* evolution experiments of WT and mutant viruses carrying different numbers of DR copies. Particularly, we deleted one, two or three copies of DR(1 + 2) from the CHIKV-Caribbean infectious clone [Δ(1 + 2)a, Δ(1 + 2)ab, Δ(1 + 2)abb’ mutants, respectively]. RNAs from WT and mutant viruses were obtained by *in vitro* transcription and transfected into BHK cells (Figure 1B). After transfection, viruses were harvested from cell culture supernatants and passaged twice in the same cell line (P1 and P2 populations). To assess the composition of the viral 3′UTR in the P2 populations, total RNA was extracted and used as a template for reverse transcription reactions with an oligo(dT) primer. The pool of cDNA was then used to amplify fragments corresponding to the CHIKV 3′UTR and cloned into the pCR2.1-TOPO vector. Finally, individual plasmid clones were analyzed by agarose gel electrophoresis and sequenced using Sanger method.

Consistent with our previous studies (35), after two passages in mammalian cells, 3′UTR-deletion variants emerged in the WT population accounting for approximately 70% of the clones analyzed. In turn, the P2 population derived from Δ(1 + 2)a mutant contained 50% of the parental virus and 50% of 3′UTR-deletion variants. For Δ(1 + 2)ab and Δ(1 + 2)abb’ mutants, only 5% and no 3′UTR-deletion variants were detected, respectively.

To extend our findings to a more physiologically relevant cell line, we transfected the WT RNA into human hepatoma Huh-7 cells and serially passaged viral populations. Importantly, deletion variants emerged in the population and accounted for approximately 40% of the clones analyzed after five viral passages (Figure 1C). Although to a different extent, the diversity of the 3′UTR in viral populations recapitulates our findings in BHK cells.

Altogether, these results indicate that the frequency of emergent viral variants carrying deletions in the 3′UTR is dependent on the number of DR copies. Populations of viruses with multiple DR copies in their 3′UTR, such as the WT virus, are mostly composed of 3′UTR-deletion variants. In contrast, populations derived from mutant viruses have less diverse 3′UTRs, suggesting that DR copies account for 3′UTR plasticity in cell culture and natural isolates.

### Infection of mosquitoes with mammalian-adapted populations

**Figure 2.**
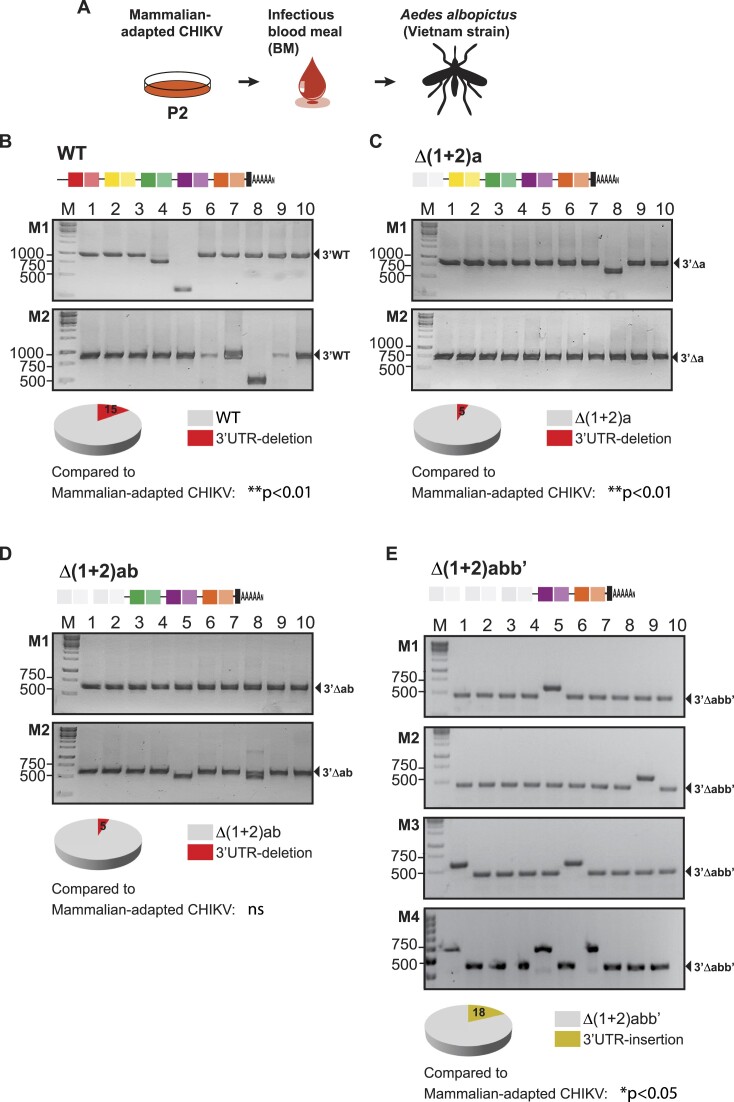
Composition of CHIKV populations after oral infection of *Aedes albopictus* mosquitoes with viruses grown in mammalian cells. (**A**) Oral infection of *Aedes albopictus* mosquitoes with WT and mutant viruses grown in mammalian cells (Figure 1). Eight days after infectious blood meal, individual mosquitoes were collected for RNA extraction and 3′UTR analysis. Eight individual mosquitoes were infected with each virus and 20 individual clones were analyzed for each mosquito. (B, C, D and E) Representative agarose gels for the 3′UTR amplification of individual clones recovered from individual mosquitoes (M1, mosquito 1; M2, mosquito 2, M3, mosquito 3 and M4, mosquito 4). The sizes of the DNA bands in the ladder (base pairs) and the parental full-length and mutant 3′UTRs are shown for reference. Below gels, pie charts for the frequencies of parental 3′UTR (gray), emergent 3′UTR-deletion variants (red) and emergent 3′UTR-insertion variants (ochre) in viral populations are shown. Statistics were performed by Fisher's exact test on cumulative data analyzing each virus in individual mosquitoes, compared to the same virus in the mammalian-adapted populations (key: ns, not significant).

To investigate how the CHIKV 3′UTR evolves in natural hosts, we used the WT or the mutant P2 populations grown in BHK cells (Figure 1B) to orally infect *Aedes albopictus* mosquitoes (Figure 2). Eight days after the infectious blood meal, we assessed the frequency of viral variants by analyzing the CHIKV 3′UTR in individual mosquitoes (Figure 2A). The frequency of the 3′UTR-deletion variants decreased from 70% to 15% for the WT virus (compare Figure 1B with Figure 2B), and from 50% to 5% for the Δ(1 + 2)a mutant (compare Figure 1B with Figure 2C). In turn, the frequency of the deletion variants did not significantly change after mosquitoes’ infection with the Δ(1 + 2)ab mutant, which contained a single DR(1 + 2) copy (compare Figure 1B with Figure 2D). These results indicate that the deletion variants that prevail in the mammalian-adapted populations are displaced within mosquitoes by others carrying longer 3′UTRs.

Strikingly, after infecting individual mosquitoes with the Δ(1 + 2)abb’ mutant, we detected new viral variants at a frequency ranging from 10% to 30% (on average 18%, Figure 2E). These emergent variants did not show 3′UTR deletions. Instead, they had longer 3′UTRs than the parental Δ(1 + 2)abb’ virus, indicating that they have incorporated blocks of sequences in their 3′UTR sequence.

**Figure 3.**
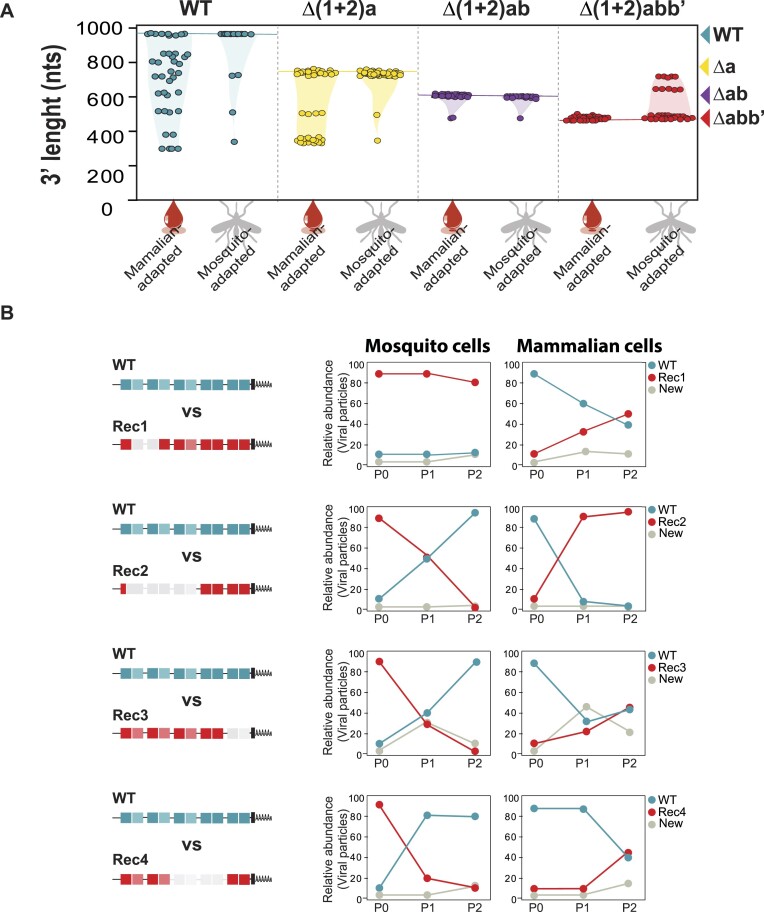
Dynamics of CHIKV populations during host switch. (**A**) Plots of CHIKV 3′UTR length of viral variants in the blood meal BM (mammalian-adapted) and eight days post-infection of individual mosquitoes (mosquito-adapted) with the WT- and mutant-derived populations. Lengths of the parental WT, Δ(1 + 2)a, Δ(1 + 2)ab and Δ(1 + 2)abb’ 3′UTRs are shown for reference. (**B**) Growth competition experiments of selected recombinant viral variants (Rec) in mammalian and mosquito cells. Mosquito (C636) and mammalian (Huh-7) cells were transfected with a mixture of WT: Rec RNAs at a 1:9 ratio and a 9:1 ratio, respectively. Viruses were harvested from the culture supernatant and used to reinfect fresh cells (P1 and P2). Left, schematic representation of the WT and the Rec viruses. For clarity, DRs are colored in turquoise and red for WT and Rec viruses, respectively, and new variants are colored in gray. Right, the relative abundance of each virus and the new emergent variants in the P0, P1 and P2 populations was assessed by resolving the cloned 3′UTR products in agarose gel electrophoresis.

The overall change in viral populations during host switching is summarized in Figure 3A. Mammalian-adapted populations of viruses initially carrying multiple DR copies are composed of 3′UTR-deletion variants that are cleared from the viral population in infected mosquitoes. In contrast, the population of viruses lacking DR copies is less diverse but results in the emergence of 3′UTR-insertion variants in mosquitoes.

Sequencing of the 3′UTR-deletion variants revealed that they contained deletions of different DR copies. To address the effect of recombinant 3′UTRs on viral replication, we cloned the 3′UTR of four selected variants into the parental backbone, performed growth competition experiments in mammalian and mosquito cells, and assessed the frequency of each virus in the P0, P1 and P2 populations (Figure 3B). In mosquito cells co-transfected with Rec and WT RNAs at a 9:1 ratio, the frequency of recombinant viruses changed from 90% to less than 10%; except for the mutant with the shortest deletion (Rec1) that remained almost unaltered. In turn, when we transfected mammalian cells with a mixture of Rec and WT RNAs at a ratio of 1:9, the recombinant viruses totally or partially displaced the WT. These results indicate that while DRs are beneficial for viral replication in mosquito cells, they have impaired fitness in mammalian cells.

Taken together, our results indicate that the evolution of the CHIKV 3′UTR during host switch is intimately linked both to the DR composition and the selective pressures imposed by each host.

### RNA recombination occurs through alternative breakpoints across the CHIKV 3′UTR

**Figure 4.**
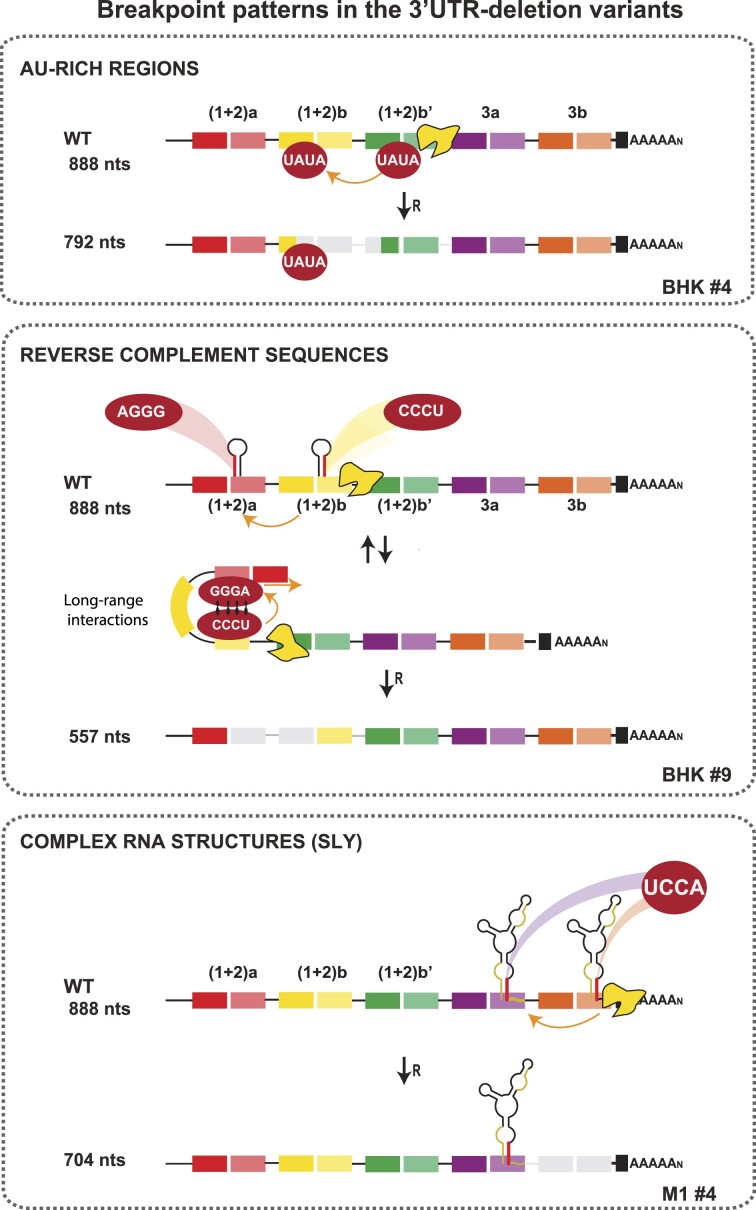
Breakpoint patterns for the 3′UTR-deletion variants in the mammalian- and mosquito-adapted populations. Representative recombination events based on the analysis of the 3′UTR-deletion variants. The letters and numbers on the bottom left correspond to those of the clones shown in Figures 1B and 2B. Three breakpoint patterns flanking the deletions were identified. Orange lines and arrows indicate the possible routes of the replication complex. Upper panel, RNA recombination guided by AU-rich regions. Middle panel, RNA recombination guided by reverse complement sequences forming hairpin-like structures or hybridizing to each other through long-range RNA–RNA interactions. Bottom panel, RNA recombination guided by a complex and highly stable RNA structure (SLY) at the DR3 end, duplicated in the CHIKV-Caribbean.

With the goal of identifying the sequences and/or structures that guide RNA recombination in the CHIKV 3′UTR, we analyzed the 3′UTR of the deletion viral variants from mammalian- and mosquito-adapted populations (Figures 1 and 2). Nucleotide sequences of the viral variants are shown in Supplementary Figure S1 and schematized in Figure 4 and Supplementary Figure S2.

The 3′UTRs of viral variants were aligned with the parental 3′UTR and the sequences flanking the deletions were examined. Our breakpoint analysis revealed that there were specific elements that guided RNA recombination in the CHIKV 3′UTR to generate the 3′UTR-deletion variants. The first consisted of adenine/uracil-rich regions in all possible combinations (Figure 4, upper panel; for more examples see Supplementary Figure S2). In particular, the sequences UAUA, AAUA, AAUU, AAAA, AAAU, AUAA and AUAU were found at the recombination breakpoints.

**Figure 5.**
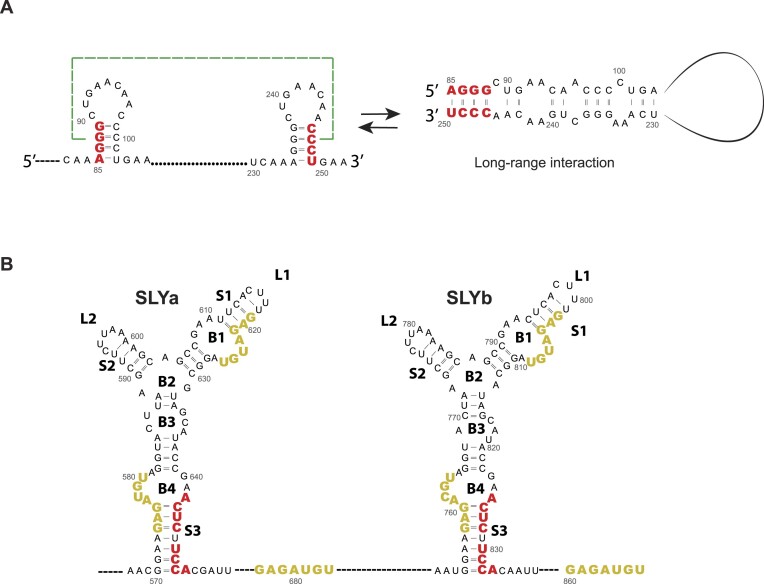
RNA secondary structures involved in RNA recombination in the CHIKV 3′UTR. (**A**) Predicted RNA secondary structure models that comprise the reverse complement sequences from the middle panel of Figure 4. The 3′UTR-deletion breakpoints are shown in red. Two mutually exclusive structures are predicted: two independent hairpin-like structures (left) and long-range RNA-RNA interactions between the reverse complement sequences (right). Numbers correspond to the nucleotide position after the translation stop codon. (**B**) Predicted RNA secondary structure model for the SLYa and SLYb, located at the 3′half of DR3. The 3′UTR-deletion breakpoints are labeled in red and the 3′UTR-deletion and -insertion breakpoints are labeled in ochre.

Other viral variants did not have repeated AU-rich sequences in the 3′UTR recombination breakpoints. Instead, they contained nucleotide sequences in reverse complement, such as the AGGG/CCCU located in the DR(1 + 2) regions (Figure 4, middle panel). Our RNA folding analysis revealed that these sequences are involved in the formation of two theoretical hairpin-like structures (Figure 5A, left panel). Alternatively, the complementary sequences that are located more than 150 nucleotides apart may be brought together through long-range RNA-RNA interactions (Figure 5A, right panel). Both RNA conformations are consistent with the SHAPE reactivity values previously published by Madden *et al.* (40), thus, further investigation is required to elucidate whether the structures responsible for RdRp template switching are the two independently folded hairpins or the long-range RNA-RNA interactions. To illustrate, we show both possibilities in the middle panel of Figure 4.

Finally, we found a third type of breakpoint pattern generated by the deletion of DR copies via RNA recombination. The UCCA, ACUC, and GAGAYGU sequences (Y = C, U or A) were identified as a recurrent breakpoint among viral variants bearing deletions that encompassed the DR3 region. According to the secondary structure prediction of the CHIKV 3′UTR, the last portion of the DR3 region folds into a highly structured Y-shaped RNA structure referred to as SLY, which is duplicated in the CHIKV-Caribbean lineage (SLYa and SLYb; Figure 4, bottom panel and Figure 5B) (42). Each SLY consists of three stems: stem 1 (S1) exposes a right loop (L1) and is interrupted by one bulge (B1); stem 2 (S2) exposes a left loop (L2) and stem 3 (S3) leaves a central bulge (B2) and is interrupted by two other bulges (B3 and B4). Interestingly, the UCCA and ACUC sequences are located in the right strand of S3 (Figure 5B, in red), while the GAGAYGU sequence is present three times in the 3′ half of each DR3 copy (Figure 5B, in ochre). The high frequency of breakpoints that mapped to the SLY suggests that the replication complex also dissociates from the template at this structure that can promote RNA recombination.

To extend our analysis, viral 3′UTR sequences were also examined in the Δ(1 + 2)a and Δ(1 + 2)ab populations. We found that, although at different rates, the mechanisms proposed for RNA recombination also occurred in the mutant viruses (Supplementary Figure S1 and Supplementary Figure S2, middle and bottom panels).

Taken together, the results indicate that different RNA patterns in the template can promote RNA recombination in the CHIKV 3′UTR, opening the possibility of manipulating the viral genome to shape the composition of viral populations.

### *In vivo* recombination at the Δ(1 + 2)abb’ 3′UTR results in the insertion of DR copies

**Figure 6.**
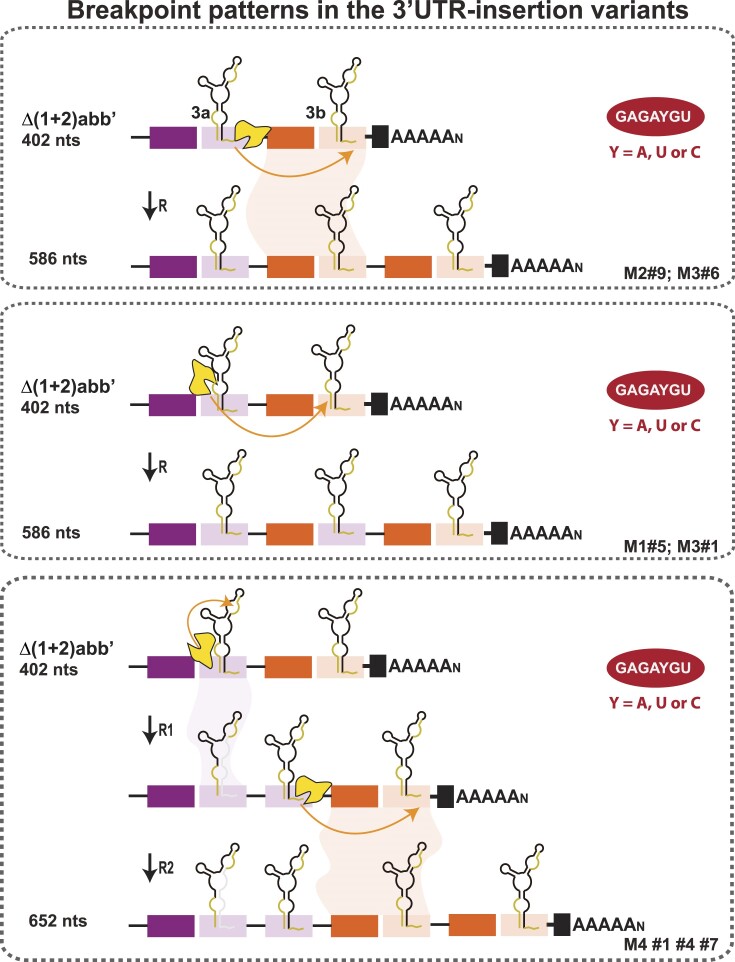
Models for the generation of 3′UTR-insertion variants in *Aedes albopictus* via RNA recombination. Representative recombination events based on the analysis of the 3′UTR-insertion variants. The letters and numbers on the bottom left correspond to the clones shown in Figure 2E. In all cases, the GAGAYGU sequence was identified at the recombination site. The locations of this sequence in each SLY is shown in ochre. Orange lines and arrows indicate the possible pathways of the replication complex. Upper panel shows template switching between the downstream sequences of the SLY. Middle panel shows switching between the S3/B4 regions. Bottom panel shows template switching in two steps: from S3/B4 to S1/B1 (step R1) and between the homologous downstream regions of SLY (step R2).

As observed, the 3′UTR of the Δ(1 + 2)abb’ population evolved in mosquitoes resulting in the emergence of 3′UTR-insertion variants that have not been detected in the mammalian-adapted populations by conventional sequencing (Figures 2E and 3). The parental Δ(1 + 2)abb’ virus lacks the three DR(1 + 2) copies but conserves a duplicated DR3 copy (DR3a and DR3b). To gain insight into the nature of the insertions, we sequenced the 3′UTR of the emergent viral variants and found that they all have gained clean copies of DR3 (Figure 6). For instance, variant #9 from Mosquito 2 (M2) and #6 from Mosquito 3 (M3) have incorporated a complete DR3b copy (in orange). In turn, variant #5 from Mosquito 1 (M1) and variant #1 from Mosquito 3 (M3) contained the duplication of the 3′ part of DR3a (in purple) and the 5′ part of DR3b (in orange) that formed together an entire DR3 copy. The three variants (#1, #4 and #7) isolated from Mosquito 4 (M4) shared the duplication of the 3′ part of DR3a (in purple) plus an entire DR3b copy (in orange). Nucleotide sequences of viral variants are shown in Supplementary Figure S3.

To identify recombination breakpoints, we aligned the 3′UTRs of the Δ(1 + 2)abb’ virus and the insertion-variants. Sequence analysis revealed that RNA recombination events involved the GAGAYGU sequence mapping in the SLY structure (Figure 5B, Figure 6, and Supplementary Figure S3, in ochre). As mentioned, the GAGAYGU sequence is present three times in the SLY structure. Indeed, it is located (i) in the S3/B4 region at the bottom of the SLY, (ii) in the S1/B1 region at the top of the SLY, and (iii) downstream of each SLY element (Figure 5B, in ochre). Based on the SLY structure, we found that while in some viral variants the replication complex switched between the downstream sequences (Figure 6, upper panel), in other viral variants it switched between the S3/B4 regions of the SLY copies (Figure 6, middle panel). In both cases, the exact SLY structure folded after a single recombination event. In turn, the Mosquito 4 viral variants (#1, #4 and #7) were likely the outcome of two independent recombination events (Figure 6, bottom panel). One event resulted from RdRp switching from S3/B4 to S1/B1 (step R1) while the other event resulted from switching between homologous downstream regions (step R2). As noted, the second event (but not the first) leads to the incorporation of a complete SLY structure.

Overall, our results showed that RNA recombination occurred in the CHIKV 3′UTR *in vivo* after infecting mosquitoes with a Δ(1 + 2)abb’-derived population. Switching of the RdRp between homologous SLY sequences provides a molecular mechanism for the generation of viruses with DR insertions.

### Insertions in the Δ(1 + 2)abb’ 3′UTR provide a fitness advantage in C6/36 cells

**Figure 7.**
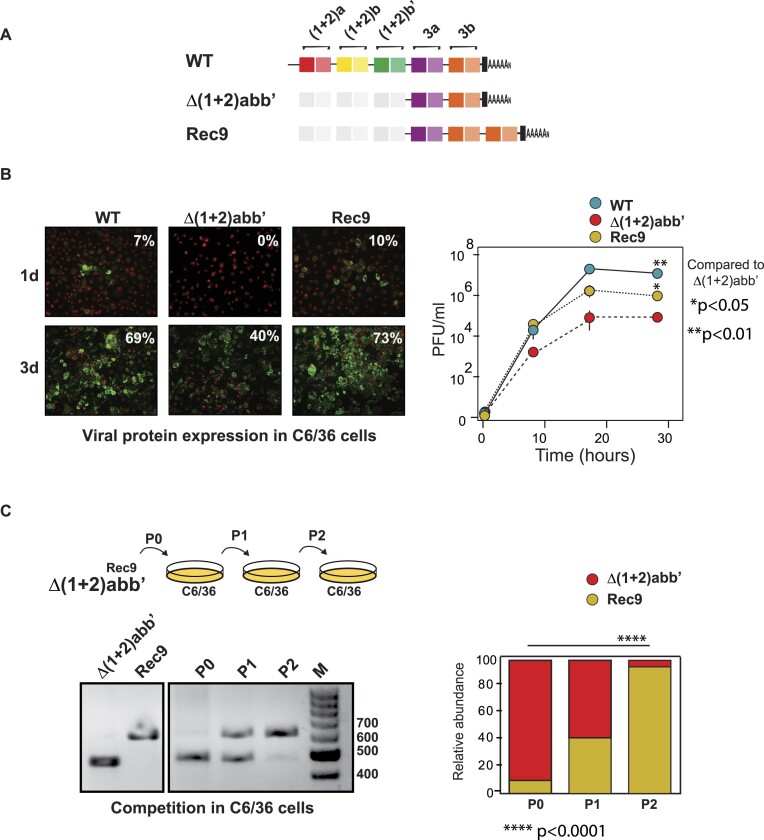
Fitness parameters of the WT virus, the Δ(1 + 2)abb’ mutant and a selected 3′UTR-insertion variant in mosquito cells. (**A**) Schematic representation of the 3′UTR of WT, Δ(1 + 2)abb’, and a variant selected in individual mosquitoes (Rec9). (**B**) Left, immunofluorescence assay at day 1 and day 3 post-transfection of Rec9 compared to WT and Δ(1 + 2)abb’ viruses. Nuclei staining with Dapi and CHIKV antigen expression are shown in red and green, respectively. Percentages of infected cells are indicated. Right, growth kinetics of the three viruses. (**C**) Growth competition experiment of the Δ(1 + 2)abb’ versus the Rec9 virus. C6/36 cells were infected with a mixture of Δ(1 + 2)abb’ and Rec9 at a 9:1 ratio (P0). Viruses were harvested from the culture supernatant and used to reinfect fresh C6/36 cells (P1 and P2). The relative abundance of each virus in the P0, P1 and P2 populations was assessed by resolving the cloned 3′UTR products in agarose gel electrophoresis and represented in the plot on the right.

Next, we asked whether insertions could compensate for the lack of DR copies providing an advantage for viral replication in mosquito cells over the Δ(1 + 2)abb’ mutant. To examine the replication of selected variants, one of the 3′UTRs cloned from a virus obtained from mosquitoes (variant #9 in M2, Figure 2E) was introduced into the parental virus, and replication parameters were evaluated in C6/36 cells. RNA transcripts from the WT, the Δ(1 + 2)abb’ or the virus containing the 3′UTR of variant #9 (Rec9) (Figure 7A) were individually transfected into C6/36 cells and virus spread was followed by viral antigen detection by immunofluorescence assay (IF) as a function of time (Figure 7B, left panel). One day post-transfection, approximately 7% of the cells in the monolayer were infected by the WT and the Rec9 viruses, while no Δ(1 + 2)abb’ infected cells were detected. At day 3 post-transfection, the WT and the Rec9 viruses infected 69% and 73% of the monolayer, respectively, compared to 40% for the Δ(1 + 2)abb’ virus at the same time point. Viral stocks were generated and titrated by plaque assays. Virus growth curves show that Rec9 did not reach WT viral titers. However, it yielded approximately 10-fold higher titers at 18 and 26 hours post-infection compared to the ΔDR(1 + 2)abb’ virus (Figure 7B, right panel), indicating that DR3 insertion partially compensates for the lack of DR(1 + 2) copies.

To better assess the viral fitness of Rec9 and ΔDR(1 + 2)abb’, growth competition experiments were performed (Figure 7C). The Δ(1 + 2)abb’ and the Rec9 viruses were mixed in a 9:1 ratio and used to infect C6/36 cells. The frequency of each virus in the P0, P1 and P2 populations was assessed by resolving the RT-PCR products encompassing the 3′UTR in agarose gel electrophoresis. We observed that the frequency of Rec9 virus markedly increased after passaging, changing from 10% in P0, to 40% in P1, and to > 90% in P2. The viral displacement demonstrates that the Rec9 virus has higher fitness than the Δ(1 + 2)abb’ mutant.

In summary, our studies suggest that RNA recombination occurs *in vivo* to maintain high fitness in a virus that must cycle between hosts.

### Recombination hotspots in the CHIKV 3′UTR

**Figure 8.**
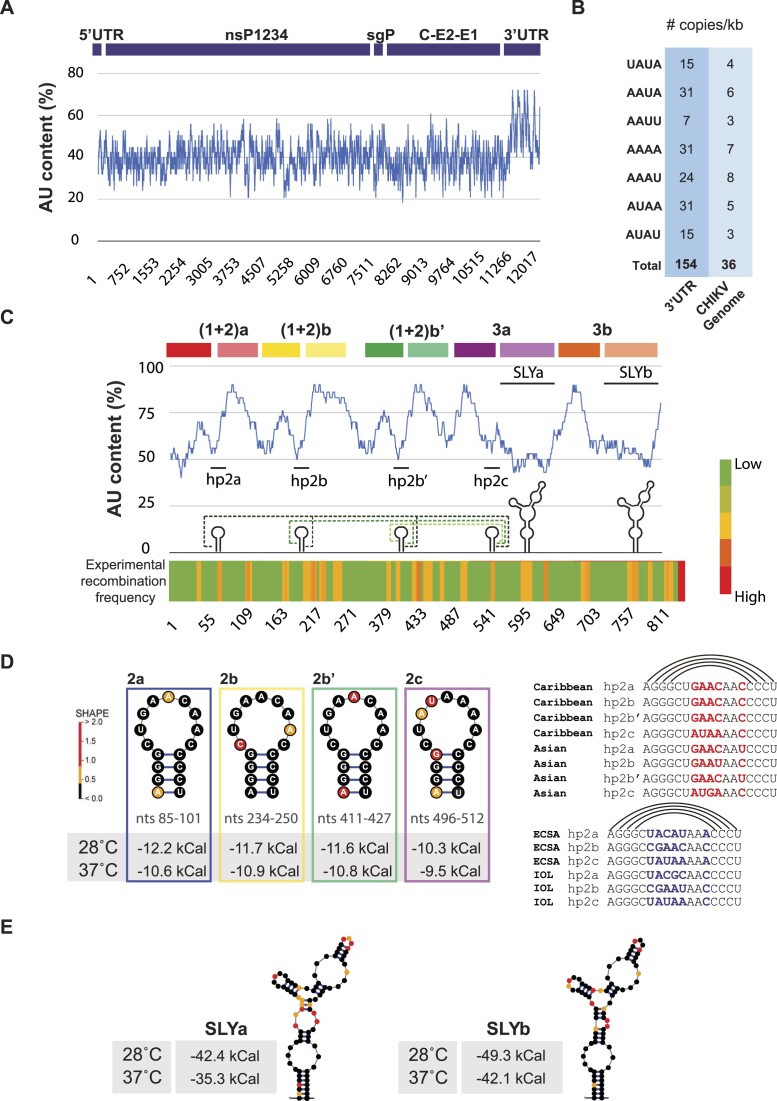
Recombination patterns in the CHIKV-Caribbean 3′UTR. (**A**) Plots of AU content as a function of the nucleotide position in the CHIKV genome, calculated as (A + U)/(A + U + G + C) × 100%. Regions corresponding to the 5′UTR, the non-structural proteins 1 to 4 (nsP1-4), the subgenomic promoter (sgP), the structural proteins (C, E2, and E1) and the 3′UTR are shown at the top. (**B**) Number of copies per kb of the different AU-rich patterns in the CHIKV genome and the 3′UTR. (**C**) Plots of AU content in the 3′UTR, calculated as before. DR blocks are shown at the top. Numbers correspond to the nucleotide position after the translation stop codon. Secondary RNA structures are schematized on the baseline and predicted long-range RNA-RNA interactions are shown with dashed lines. Bottom, heat map illustrating the frequency of recombination breakpoints from our adaptation experiments in mammalian cells and mosquitoes. (**D**) SHAPE-constrained thermodynamic model for the individual hairpin-like structures (hp2a, hp2b, hp2b’ and hp2c) formed by sequences in reverse complement across the CHIKV 3′UTR at 28°C and 37°C. Minimum free energies at both temperatures are shown. Right, sequence alignments and nucleotide conservation of the region that folds into harpin-like structures for representative Caribbean, Asian, ECSA, and IOL (Indic Ocean) lineages. Conserved nucleotides are in black, and variable nucleotides are in red and blue. Arcs indicate the nucleotide pairings that form the stem of each harpin-like structure. (**E**) SHAPE-constrained thermodynamic model for SLYa and SLYb at 28°C and 37°C, with corresponding minimum free energies.

To investigate the abundance of RNA patterns that promote recombination in the 3′UTR, we first examined the AU content of the CHIKV genome. Interestingly, we found that it is significantly higher in the CHIKV 3′UTR than in other regions of the viral genome (Figure 8A, upper panel). For comparison, the AU content in the CHIKV-Caribbean 3′UTR (calculated as (A + U)/(A + U + G + C) × 100) represents 67% of the nucleotides, while in the 5′UTR it is 55%, and in the subgenomic promoter region, 44%. To extend the study, we analyzed the abundance of the UAUA, AAUA, AAUU, AAAA, AAAU, AUAA and AUAU motifs and found that they are overrepresented in the 3′UTR compared to the rest of the genome (Figure 8B). Next, we analyzed the AU content of the 3′UTR and found that the SLY and the hairpin structures are composed of only 55% AU (Figure 8C). This suggests that the AU surplus in the 3′UTR is unlikely to be involved in the formation of RNA secondary structures. Based on these observations, we speculate that unstructured AU-rich regions in the 3′UTR favor the dissociation of the replication complex that reassociates to a homologous AU-rich region in either the same or a different RNA template to resume RNA synthesis. The resulting 3′UTR has the deletion of one copy of the AU-rich region plus the deletion of the nucleotide sequences between the two copies.

Yet another trait for RNA recombination was the presence of secondary RNA structures. Interestingly, the CHIKV 3′UTR contains several hairpin-like structures that are conserved among viral lineages (Figure 8D). Despite subtle differences in minimum free energies, identical RNA structures are formed at 28°C and 37°C, suggesting that the thermodynamics of folding are conserved at the infection temperature of mammals and mosquitoes, respectively. Moreover, all hairpins are maintained by C/G interactions but contain different sequences in the loops (in bold), suggesting that the structure is relevant regardless of the loop sequences.

Furthermore, complex RNA structures such as the SLY were likely to promote RNA recombination and precisely, we found that UCCA, ACUC, and GAGAYGU sequences were recurrent recombination breakpoints (Figure 5). Interestingly, the GAGAYGU copies are in the junctions between DR blocks (Figure 8C), suggesting that this sequence may have been involved in the incorporation of DR3 copies when CHIKV moved from Africa to Asia during natural evolution. As in the case of hairpin structures, SLYs equally fold at 28°C and 37°C (Figure 8E), suggesting that they might favor template switching in both the mammalian host and the mosquito vector.

To experimentally evaluate the effect of temperature on recombination frequencies, we cultured the WT virus in mammalian cells at 28°C and 37°C and analyzed the 3′UTR of the adapted viral variants (Supplementary Figure S4). After two viral passages in BHK cells, deletion variants accounted for 51% of the clones analyzed at 28°C, not significantly different from their abundance at 37°C.

In Huh-7 cells, deletion variants accounted for about 42% after five viral passages at the two temperatures. These results suggest that differences in the composition of viral populations grown in mosquito and mammalian cells are likely due to host-specific selective pressures on the 3′UTR, rather than differences in growth temperature.

To visualize the recombination hotspots across the 3′UTR, we generated a heat map based on the frequency of the breakpoints that were identified from *in vitro* and *in vivo* evolution experiments (Figure 8C, bottom). As noted, RNA recombination occurred preferentially in defined regions of the 3′UTR, particularly, where AU content peaks and where there are RNA structural elements.

### RNA motifs in the CHIKV 3′UTR as genetic determinants of recombination

**Figure 9.**
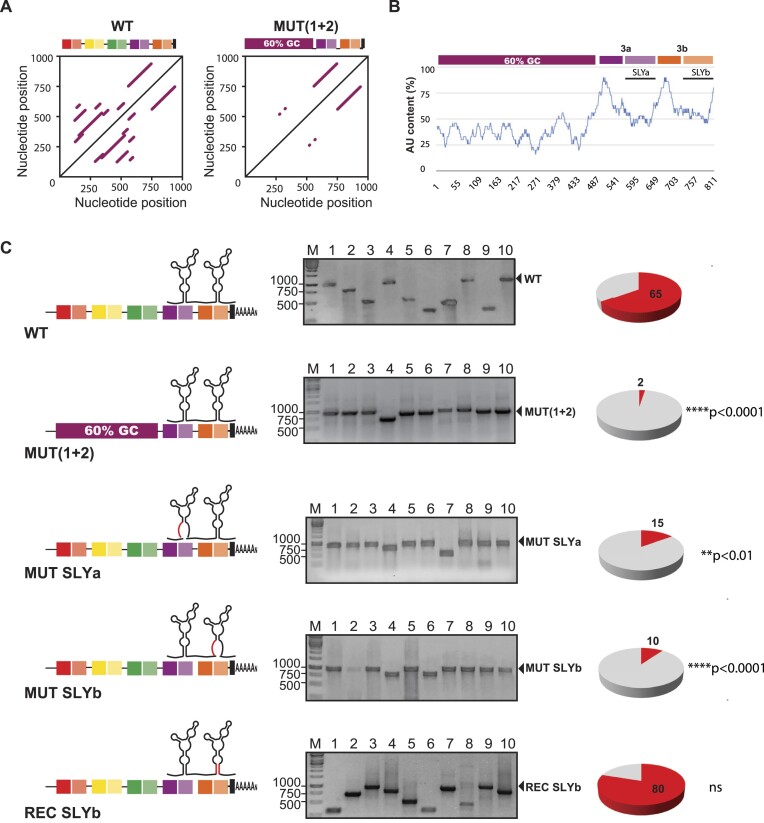
Role of RNA sequences and structures on CHIKV 3′UTR evolution. (**A**) RNA matrix comparison of the WT 3′UTR and the 3′UTR of an engineered mutant, where DR (1 + 2) was replaced by a 60% GC non-repeating sequence. Lines parallel to the central diagonal line evidence sequence repeats. (**B**) Plot of AU content as a function of the nucleotide position in the Mut DR(1 + 2) 3′UTR. (**C**) Left, schematic representations of the 3′UTRs of the WT virus and Mut (1 + 2) and mutant viruses with the disruption of one or the other SLY copy (Mut SLYa and Mut SLYb) or the reconstitution of SLYb (Rec SLYb). Middle, representative agarose gels for PCR amplification of the 3′UTRs of individual clones recovered from WT and mutant viral populations passaged twice in BHK cells. Right, pie charts for the frequencies of parental 3′UTR (gray) and emergent 3′UTR-deletion variants (red) in viral populations. Statistics were performed using Fisher's exact test on cumulative data analyzing each condition vs. the WT.

To conclusively demonstrate the effect of sequence repeats on the composition of viral populations, we exchanged the DR(1 + 2) copies for an unrelated sequence that had no sequence repeats and 60% GC content [Mut DR(1 + 2), Figure 9A and B]. We synthesized viral genomic RNA molecules and transfected them in BHK cells. We then performed two successive viral passages and analyzed the 3′UTRs in the P2 population (Figure 9C). Consistent with data presented in Figure 1, the WT-derived population contained 65% new viral variants. In contrast, the Mut (1 + 2)-derived population contained only 2% new viral variants. This result indicates that the mutant is restricted for 3′UTR evolution, despite having intact SLYs.

Next, we investigated the role of SLY structures. To this end, we introduced point mutations that altered the SLYa or SLYb structures by incorporating mismatches in the left strand of S3 (respectively, Mut SLYa and Mut SLYb). After two passages in BHK cells, we observed that recombinant viral variants represented 15% and 10% in the Mut SLYa- and Mut SLYb-derived populations, respectively. To demonstrate that the SLY’s function depends on the structure and not on the conservation of the nucleotide sequences that paired at the base of S3, we designed an additional viral mutant carrying intact SLYs but with different nucleotide sequences (Rec SLYb). Remarkably, the abundance of recombinant viral variants in the Rec SLYb population was 80%, comparable to the WT.

Altogether, our reverse genetic study indicates that, albeit at low frequencies, RNA recombination happens regardless of an intact SLY element or sequence repeats. This is due to the presence of multiple traits that favor template switching in the CHIKV 3′UTR. However, disruption of SLY structures in DR3 or replacement of DR(1 + 2) by non-repeating sequences, results in a much less diverse mutant spectrum in the virus population compared to the WT, in support of a pivotal role of all conserved 3′RNA motives in virus evolution.

Overall, our findings provide a new insight to understand the molecular basis of RNA recombination as a key mechanism modulating CHIKV evolution and host-adaptation.

## Discussion

This work describes the dynamics of CHIKV populations *in vitro* in mammalian cells and *in vivo* in laboratory mosquitoes’ strains. We found that the 3′UTR of the CHIKV genome has a host-adaptable architecture with multiple DR copies that are conserved due to demands for viral replication in mosquitoes. To sustain high viral fitness in the vertebrate host and the mosquito vector, cycles of loss and gain of DR copies occur in the CHIKV 3′UTR during host switching. RNA recombination provides the means to add or remove DR blocks, building a customized 3′UTR that is functional in each host. In mosquitoes experimentally infected with a CHIKV population with impaired transmission rate, the addition of DR blocks leads to the emergence of new viral variants that restore viral fitness (Figures 6 and 7). Although opposite selective pressures have been observed in the CHIKV 3′ UTR during mammalian versus mosquito infections *in vitro* (31,34,37) and *in vivo* (38,43,44), further investigation is required to elucidate the precise role of sequence repeats.

Recombination breakpoints have been the object of study for many RNA viruses. In accordance with our observations, breakpoints have been found to cluster near AU-rich regions (45) and next to stem-loop structures (46–48) in other RNA viruses, suggesting that these are pervasive features that guide viral RNA recombination. A plausible explanation for the AU abundance close to breakpoints relies on the fact that the RNA–RNA interaction between the nascent strand and the template is weaker when maintained by A/U hybridization, which would favor RdRp dissociation. In turn, RNA structural elements might promote RNA recombination by inhibiting the movement of RdRp. Since both substitution of sequence repeats and disruption of SLY structures reduce the diversity of viral populations, it is likely that these RNA elements must act in concert to ensure adequate rates of recombination in the CHIKV 3′UTR.

Based on our estimation of minimum free energy values (Figure 8D and E), RNA structures appear to be subtly more stable at mosquito temperature than at mammalian temperature. However, the abundance of recombinant viral variants was higher in mammalian cells (Figure 3A). Hence, we consider it most plausible that the distinct spectrum of viral populations grown in insect and mammalian cells is due to host-specific selective pressures (Figure 3B) rather than to differences in recombination frequency.

Although there are many traits capable of promoting RNA recombination in the CHIKV 3′UTR via diverse molecular mechanisms, accumulated evidence suggests that the basis that governs template switching is likely common to different regions of the viral genome, viral families, and host environments.

Uncovering the mechanisms of RNA recombination can help to understand the evolution of CHIKV and the emergence of epidemic lineages. In this regard, sequence analyses from our adaptation experiments using DR-deletion mutants suggest a clear association between the organization of the CHIKV 3′UTR and the variant spectrum in viral populations (Figure 1). Considering that ongoing CHIKV epidemics are caused by viral strains that differ substantially in their 3′UTR organization, it is likely that epidemic viruses also vary in their evolutionary potential. For instance, the CHIKV-Caribbean loses DR copies during adaptation to mammalian cells but retains a long 3′UTR in natural isolates, probably due to requirements for viral replication in mosquitoes. Unlike the CHIKV-Caribbean 3′UTR, the 3′UTR from the CHIKV responsible for the 2005–2006 epidemic in La Réunion Island that belongs to the Indian Ocean linage (IOL), carries only two copies of DR1 and three copies of DR2. We have previously observed that La Réunion 3′UTR is much more stable in cell culture than the Caribbean 3′UTR (35), supporting our hypothesis about the relevance of DR copies for viral evolution.

Besides being modulated by RNA elements, RNA recombination is influenced by RdRp fidelity (49,50) and a combination of host and environmental factors (51,52). However, how the interplay between viral and host factors drives CHIKV evolution requires further investigation. Identifying the viral and host contributions for RNA recombination can help to modulate the recombination rate with different goals. On the one hand, recombination-deficient viruses are currently being developed as vaccine candidates to prevent reversion to virulence (53). On the other hand, enhancement of RNA recombination yields defective viral genomes (DVGs), which have antiviral effects and strong immunostimulatory potential (54). For these reasons, DVGs are being evaluated as candidates for human therapeutics and vector control strategies to mitigate arbovirus transmission and disease (55,56). Collectively, the evidence to date supports the notion that RNA recombination should be maintained in balance to sustain efficient viral replication and virulence.

A thorough understanding of the intricacies of RNA recombination could be used to design genetically engineered viruses, opening new avenues for the rational development of vaccines.

## Concluding remarks

We found that viral populations are composed of variants carrying DR deletions in the 3′UTR, which are generated by RNA recombination in vertebrate hosts. Diversity of viral populations depends on the organization of the CHIKV 3′UTR, suggesting that the DR copies are involved in the recombination process. Within mosquitoes, deletion variants are cleared out and fitter insertion variants emerge after the infection with a virus carrying large DR deletions in the 3′UTR. These results strengthen the idea that RNA recombination in the CHIKV 3′UTR acts as a strategy to overcome genetic bottlenecks during cycling. Moreover, we identified alternative breakpoint patterns generated by template switching in the CHIKV 3′UTR, gaining insight into the molecular mechanism that has been associated with the re-emergence of CHIKV epidemic strains.

## Supplementary Material

gkae650_Supplemental_Files

## Data Availability

The data underlying this article are available in the article and in its online supplementary material.
