# Supplementary material for: Molecular basis of RNA recombination in the 3′UTR of chikungunya virus genome
Source: Nucleic Acids Res. 2024 Jul 25;52(16):9727–44. doi: 10.1093/nar/gkae650 (PMC11381336; doi:10.1093/nar/gkae650)
Supplement: gkae650_Supplemental_Files [file gkae650_supplemental_files.zip › Supplementary data NAR-02816-R-2023.pdf]

S1 FIGURE

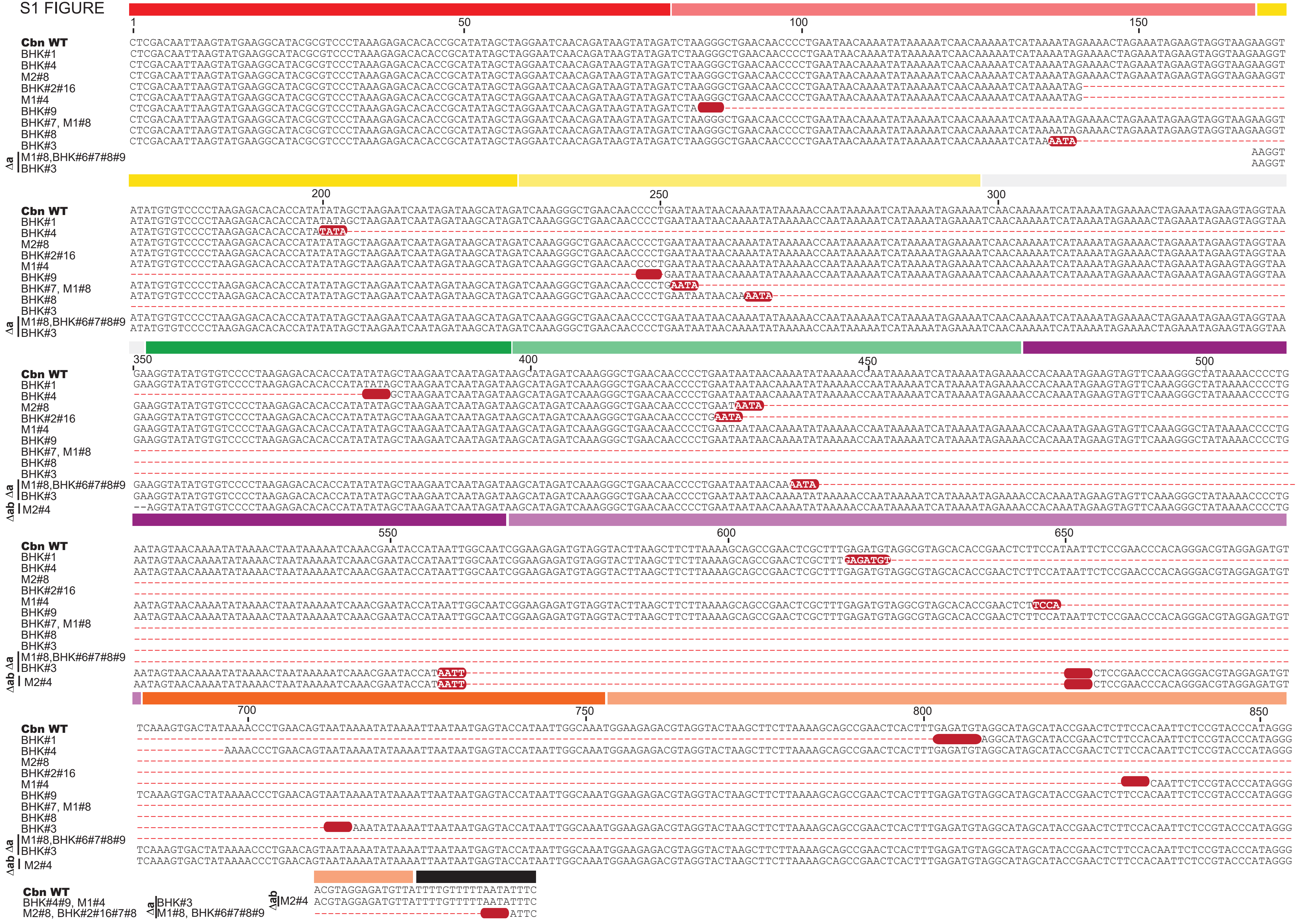
1
50
100
150
200
250
300
350
400
450
500
550
600
650
700
750
800
850
Cbn WT
BHK#1
BHK#4
M2#8
BHK#2#16
M1#4
BHK#9
BHK#7, M1#8
BHK#8
BHK#3
Δa
M1#8,BHK#6#7#8#9
BHK#3
Δab
M2#4
BHK#4#9, M1#4
M2#8, BHK#2#16#7#8
M1#8, BHK#6#7#8#9
AATA
TATA
GAGATGT
TCCA
AATT
ACGTAGGAGATGTTATTTTGTTTTTAATATTTC
ATTC

S1 Fig. Nucleotide sequences of the 3'UTR-deletion variants in the WT or mutant populations adapted to mammalian cells or *Aedes albopictus* mosquitoes. Alignment of nucleotide sequences corresponding to the 3'UTR of the WT, Δ(1+2)a, Δ(1+2)ab, and Δ(1+2)abb' viral populations after two passages (P2) in BHK cells (Fig 1) or eight days post-infection of *Aedes albopictus* mosquitoes (Fig 2). The input CHIKV-Caribbean sequence is presented as reference. The numbers on the left correspond to clones shown in Fig 1B and Fig 2B, Fig 2C and Fig 2D, Fig 4, Fig 6 and Fig S2. Recombination breakpoints are indicated with red ovals. Position 1 refers to the first position after the translation stop codon.

S2 FIGURE

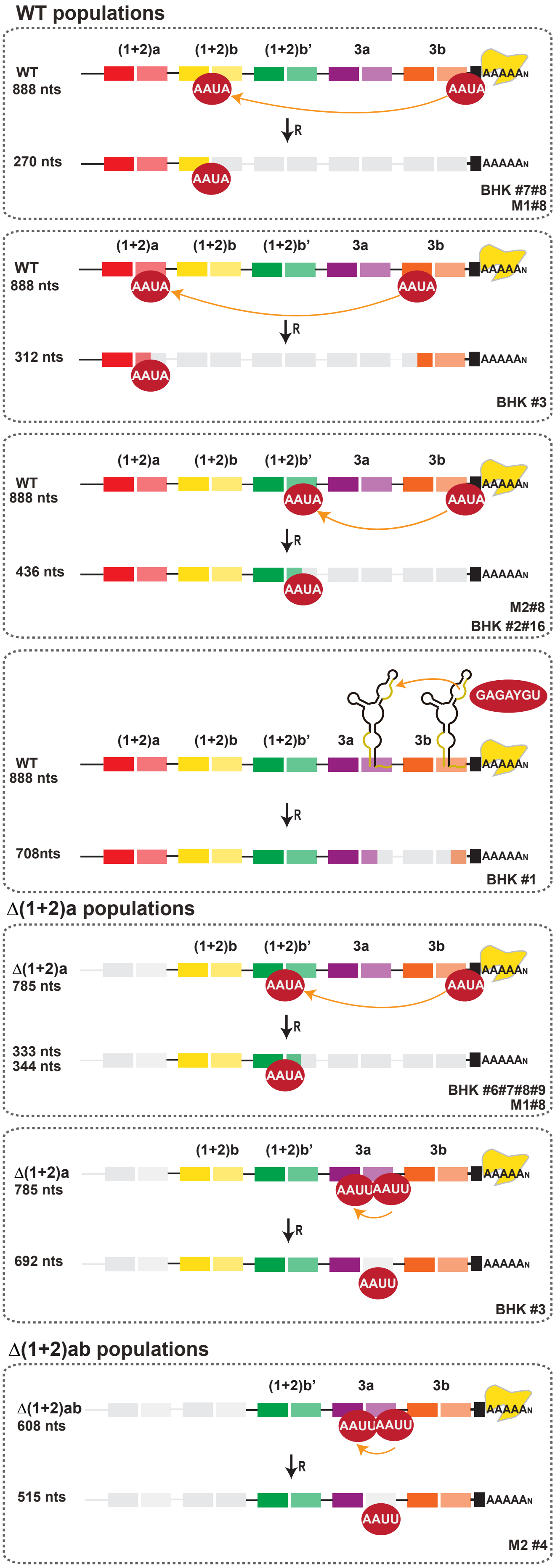

S2 Fig. Breakpoint patterns for the 3'UTR-deletion variants in the WT, Δ(1+2)a, and Δ(1+2)ab derived populations. The 3'UTRs of additional deletion variants to those in Fig 1 and Fig 2 are schematized. Recombination breakpoints are indicated with red ovals. Upper panel, 3'UTR-deletion variants in the WT population. Middle panel, a 3'UTR-deletion variant in the Δ(1+2)a population. Bottom panel, a 3'UTR-deletion variant in the Δ(1+2)ab population. Orange lines and arrows indicate the possible routes of the replication complex.

S3 FIGURE

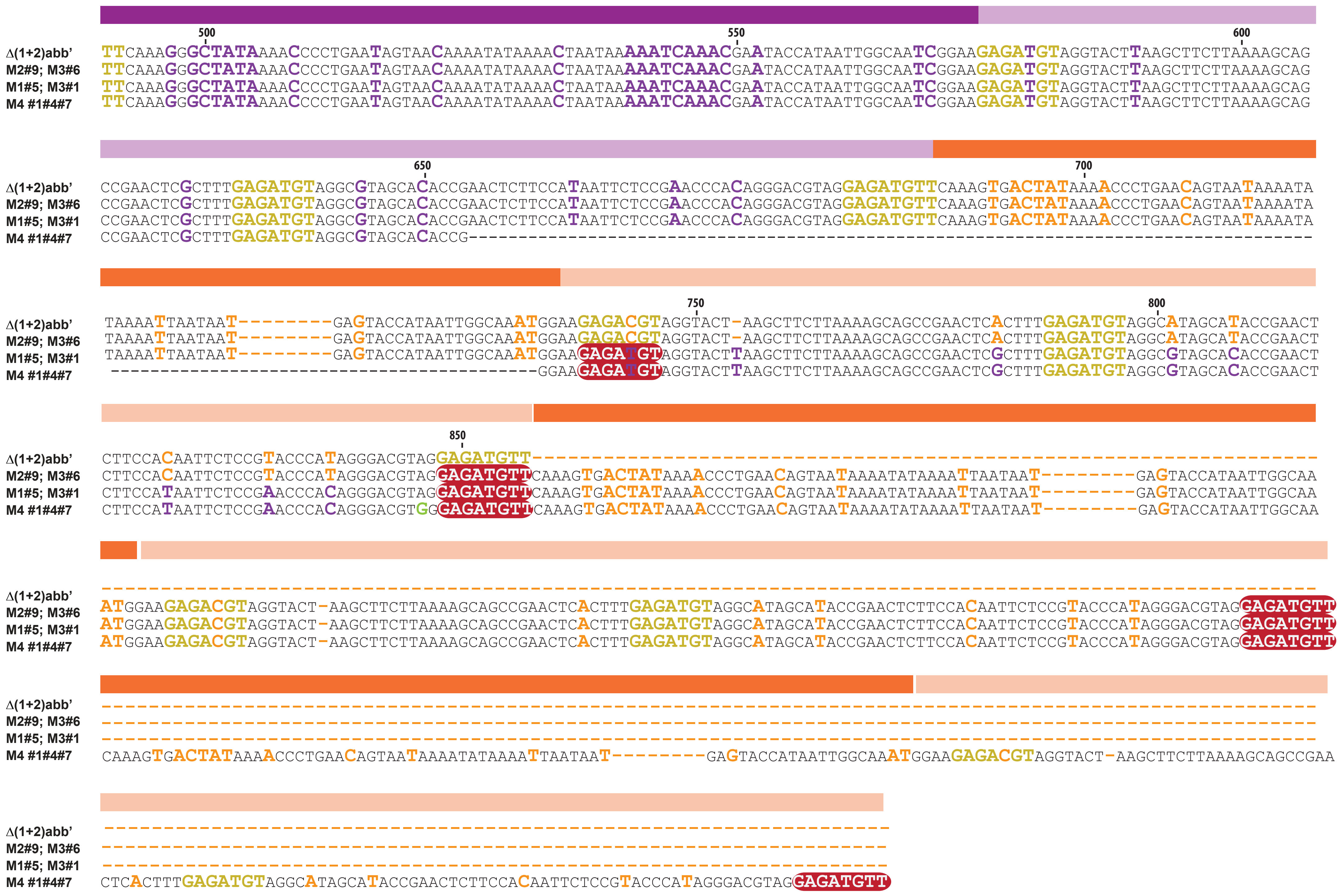

500
550
600
Δ(1+2)abb' TTCAAAGGGCTATAAAACCCCTGAATAGTAACAAAATATAAAACTAATAAAAATCAAACGAATACCATAATTGGCAATCGGAAGAGATGTAGGTACTTAAGCTTCTTAAAAGCAG
M2#9; M3#6 TTCAAAGGGCTATAAAACCCCTGAATAGTAACAAAATATAAAACTAATAAAAATCAAACGAATACCATAATTGGCAATCGGAAGAGATGTAGGTACTTAAGCTTCTTAAAAGCAG
M1#5; M3#1 TTCAAAGGGCTATAAAACCCCTGAATAGTAACAAAATATAAAACTAATAAAAATCAAACGAATACCATAATTGGCAATCGGAAGAGATGTAGGTACTTAAGCTTCTTAAAAGCAG
M4 #1#4#7 TTCAAAGGGCTATAAAACCCCTGAATAGTAACAAAATATAAAACTAATAAAAATCAAACGAATACCATAATTGGCAATCGGAAGAGATGTAGGTACTTAAGCTTCTTAAAAGCAG
650
700
Δ(1+2)abb' CCGAACTCGCTTTGAGATGTAGGCGTAGCACACCGAACTCTTCCATAATTCTCCGAACCCACAGGGACGTAGGAGATGTTCAAAGTGACTATAAAACCCTGAACAGTAATAAAATA
M2#9; M3#6 CCGAACTCGCTTTGAGATGTAGGCGTAGCACACCGAACTCTTCCATAATTCTCCGAACCCACAGGGACGTAGGAGATGTTCAAAGTGACTATAAAACCCTGAACAGTAATAAAATA
M1#5; M3#1 CCGAACTCGCTTTGAGATGTAGGCGTAGCACACCGAACTCTTCCATAATTCTCCGAACCCACAGGGACGTAGGAGATGTTCAAAGTGACTATAAAACCCTGAACAGTAATAAAATA
M4 #1#4#7 CCGAACTCGCTTTGAGATGTAGGCGTAGCACACCG--
750
800
Δ(1+2)abb' TAAAATTAATAAT---------GAGTACCATAATTGGCAAATGGAAGAGACGTAGGTACT-AAGCTTCTTAAAAGCAGCCGAACTCACTTTGAGATGTAGGCATAGCATACCGAACT
M2#9; M3#6 TAAAATTAATAAT---------GAGTACCATAATTGGCAAATGGAAGAGACGTAGGTACT-AAGCTTCTTAAAAGCAGCCGAACTCACTTTGAGATGTAGGCATAGCATACCGAACT
M1#5; M3#1 TAAAATTAATAAT---------GAGTACCATAATTGGCAAATGGAAGAGATGTAGGTACTTAAGCTTCTTAAAAGCAGCCGAACTCGCTTTGAGATGTAGGCGTAGCACACCGAACT
M4 #1#4#7 ---GGAAGAGATGTAGGTACTTAAGCTTCTTAAAAGCAGCCGAACTCGCTTTGAGATGTAGGCGTAGCACACCGAACT
850
Δ(1+2)abb' CTTCCACAATTCTCCGTACCCATAGGGACGTAGGAGATGTT---
M2#9; M3#6 CTTCCACAATTCTCCGTACCCATAGGGACGTAGGAGATGTTCAAAGTGACTATAAAACCCTGAACAGTAATAAAATATAAAATTAATAAT---------GAGTACCATAATTGGCAA
M1#5; M3#1 CTTCCATAATTCTCCGAACCCACAGGGACGTAGGAGATGTTCAAAGTGACTATAAAACCCTGAACAGTAATAAAATATAAAATTAATAAT---------GAGTACCATAATTGGCAA
M4 #1#4#7 CTTCCATAATTCTCCGAACCCACAGGGACGTGGGAGATGTTCAAAGTGACTATAAAACCCTGAACAGTAATAAAATATAAAATTAATAAT---------GAGTACCATAATTGGCAA
Δ(1+2)abb' --
M2#9; M3#6 ATGGAAGAGACGTAGGTACT-AAGCTTCTTAAAAGCAGCCGAACTCACTTTGAGATGTAGGCATAGCATACCGAACTCTTCCACAATTCTCCGTACCCATAGGGACGTAGGAGATGTT
M1#5; M3#1 ATGGAAGAGACGTAGGTACT-AAGCTTCTTAAAAGCAGCCGAACTCACTTTGAGATGTAGGCATAGCATACCGAACTCTTCCACAATTCTCCGTACCCATAGGGACGTAGGAGATGTT
M4 #1#4#7 ATGGAAGAGACGTAGGTACT-AAGCTTCTTAAAAGCAGCCGAACTCACTTTGAGATGTAGGCATAGCATACCGAACTCTTCCACAATTCTCCGTACCCATAGGGACGTAGGAGATGTT
Δ(1+2)abb' --
M2#9; M3#6 --
M1#5; M3#1 --
M4 #1#4#7 CAAAGTGACTATAAAACCCTGAACAGTAATAAAATATAAAATTAATAAT---------GAGTACCATAATTGGCAAATGGAAGAGACGTAGGTACT-AAGCTTCTTAAAAGCAGCCGAA
Δ(1+2)abb' --
M2#9; M3#6 --
M1#5; M3#1 --
M4 #1#4#7 CTCACTTTGAGATGTAGGCATAGCATACCGAACTCTTCCACAATTCTCCGTACCCATAGGGACGTAGGAGATGTT

S3 Fig. Nucleotide sequences of the 3'UTR-insertion variants that emerged in the Δ(1+2)abb' population after the infection of *Aedes albopictus*. Alignment of nucleotide sequences corresponding to the 3'UTR of the insertion variants, eight days after the infection of *Aedes* mosquitoes with the Δ(1+2)abb' virus grown in BHK cells. The input Δ(1+2)abb' CHIKV-Caribbean sequence is shown as a reference. The numbers on the left correspond to clones schematized in Fig 2E. Recombination breakpoints are indicated with red ovals. Position 1 refers to the first position after the translation stop codon in the WT CHIKV-Caribbean. Nucleotides in DR3a that differ from those at the same position in DR3b are labeled in orange. Nucleotides in DR3b that differ from those at the same position in DR3b are labeled in purple.

FIGURE S4

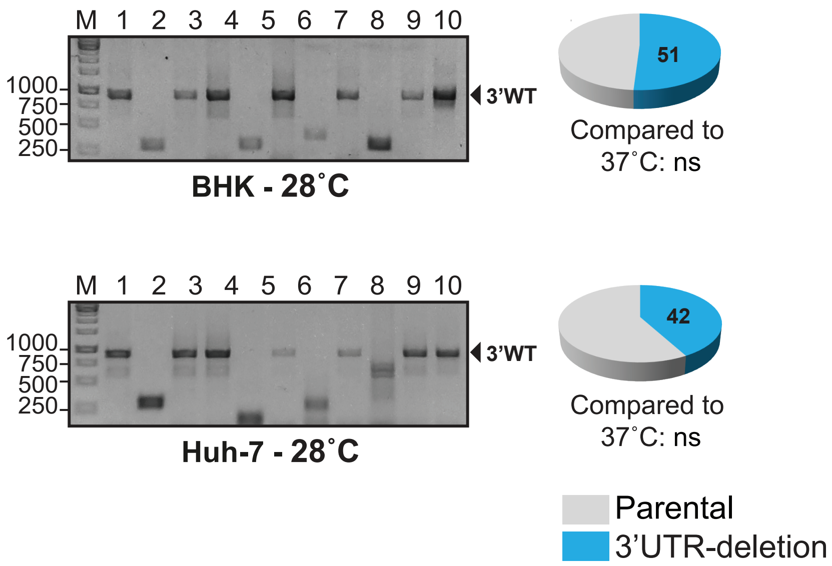

Fig S4. Composition of CHIKV WT populations grown at 28°C in mammalian cells. BHK and Huh-7 were transfected with the WT RNA and viruses were successively passaged at 28°C in the same cells. Two independent *in vitro* evolution experiments were performed, and 20 individual clones were analyzed for each one. Left, representative agarose gels for PCR amplification of the 3'UTRs of individual clones. The sizes of DNA bands in the ladder (base pairs) and the WT 3'UTR are shown for reference. Right, pie charts for the frequencies of WT 3'UTR (gray) and emergent 3'UTR-deletion variants (blue) in viral populations. Statistics were performed using Fisher's exact test on cumulative data analyzing 28°C vs. 37°C in the same cell line (Key: ns, not significant).

SUPPLEMENTARY TABLE 1

	Cloning approach	First PCR		Second PCR	
		Primers	Template	Destination Plasmid	Description
Δ(1+2)a<br><br>Δ(1+2)ab	Cloning between unique *Sac*I and *Not*I restriction sites in CHIKV-Cbn	**118** 5'TAAGAGCTCTACATAAATAGGTATAC3'<br>**92** 5'CGAAACAAGCGCTCATGAGC3'			Deletion of DR(1+2)a or Δ(1+2)ab copies.
Δ(1+2)abb'	Cloning between unique *Sac*I and *Not*I restriction sites in CHIKV-Cbn *Sac*I	**96** 5'GCACTAAGAGCTCGTTCAAAGGGCTATAAAACCCC3'<br>**92** 5'CGAAACAAGCGCTCATGAGC3'			Deletion of DR(1+2)abb' copies.
Mut SLYa<br><br>Mut SLYb	Overlapping PCR and cloning between unique *Sac*I and *Not*I restriction sites in CHIKV-Cbn	OL fragment 1:<br>**154** 5'CAATCGCTTCTCATGTAGGTACTTAAGCTTC3'<br>**92** 5'CGAAACAAGCGCTCATGAGC3'<br>OL fragment 2:<br>**94** 5'-TCAGCAGGCACTAAGAGCTCGACAATTAAGTA-3'<br>**155** 5'CTACATGAGAAGCGATTGCCAATTATGGTA3'			Disruption of SLYa or SLYb structures.
Rec SLYb	Restriction-free	**SV30** 5'GTACGGAGAATTGTGCTTCTCTTCGGTATGCTAT3'<br>**94** 5'TCAGCAGGCACTAAGAGACTCGACAATTAAGTA3'	Mut SLYb	Mut SLYb	Reconstitution of SLYb structure.
CHIKV Cbn *BamH*I	Restriction-free	**94** 5'TCAGCAGGCACTAAGAGACTCGACAATTAAGTA3'<br>**SV31** 5'GTTTTATAGCCCTTTGGAtCCTACTTCTATTTGTGGTT3'		CHIKV-Cbn *Sac*I	Construction of a *Sac*I-*BamH*I cloning cassette
Mut (1+2)	Cloning between *Sac*I and *BamH*I restriction sites in the CHIKV-Cbn	**SV34** 5'CCGCTCGGAGGAGAGCTCCCAGCCCATGGTC3'<br>**SV35** 5'GACCGGCTGAAAAGCCTGATC3'	firefly-mCherry plasmid	CHIKV-Cbn *Sac*I *BamH*I	Replacement of DR(1+2) copies by a non-related 60%GC sequence.
Recombinant variants	Cloning between unique *Sac*I and *Not*I restriction sites in CHIKV-Cbn	**116** 5'CTAATCGTGGTGCTATGC3'<br>**122** 5'TTAGCGGCCGCTTTTTTTTTTTTTTTTTTTTTTTTTGAAATAT3'			Introduction of the 3'UTR of selected variants into CHIKV-Cbn
