# Supplementary material for: Molecular basis of RNA recombination in the 3′UTR of chikungunya virus genome
Source: Nucleic Acids Res. 2024 Jul 25;52(16):9727–44. doi: 10.1093/nar/gkae650 (PMC11381336; doi:10.1093/nar/gkae650)
Supplement: gkae650_Supplemental_Files [file gkae650_supplemental_files.zip › SUPPLEMENTARY TABLE S1.docx]

SUPPLEMENTARY TABLE S1

|  | Cloning approach | First PCR | | Second PCR |  |
| --- | --- | --- | --- | --- | --- |
|  |  | Primers | Template | Destination Plasmid | Description |
| Δ(1+2)a    Δ(1+2)ab | Cloning between unique *Sac*I and *Not*I restriction sites in CHIKV-Cbn | **118** 5’TAAGAGCTCTACATAAATAGGTATAC3’  **92** 5’CGAAACAAGCGCTCATGAGC3’ |  |  | Deletion of DR(1+2)a or Δ(1+2)ab copies. |
| Δ(1+2)abb’ | Cloning between unique *Sac*I and *Not*I restriction sites in CHIKV-Cbn *Sac*I | **96** 5’GCACTAAGAGCTCGTTCAAAGGGCTATAAAACCCC3’  **92** 5’CGAAACAAGCGCTCATGAGC3’ |  |  | Deletion of DR(1+2)abb’ copies. |
| Mut SLYa  Mut SLYb | Overlapping PCR and cloning between unique *Sac*I and *Not*I restriction sites in CHIKV-Cbn | OL fragment 1:  **154** 5’CAATCGCTTCTCATGTAGGTACTTAAGCTTC3’  **92** 5’CGAAACAAGCGCTCATGAGC3’  OL fragment 2:  **94** 5’-TCAGCAGGCACTAAGAGCTCGACAATTAAGTA-3’  **155** 5’CTACATGAGAAGCGATTGCCAATTATGGTA3’ |  |  | Disruption of SLYa or SLYb structures. |
| Rec SLYb | Restriction-free | **SV30** 5’GTACGGAGAATTGTGCTTCTCTTCGGTATGCTAT3’  **94** 5’TCAGCAGGCACTAAGAGACTCGACAATTAAGTA3’ | Mut SLYb | Mut SLYb | Reconstitution of SLYb structure. |
| CHIKV Cbn *BamH*I | Restriction-free | **94** 5’TCAGCAGGCACTAAGAGACTCGACAATTAAGTA3’  **SV31** 5’GTTTTATAGCCCTTTGGAtCCTACTTCTATTTGTGGTT3’ |  | CHIKV-Cbn *Sac*I | Construction of a *Sac*I-*BamH*I cloning cassette |
| Mut (1+2) | Cloning between *Sac*I and *BamH*I restriction sites in the CHIKV-Cbn | **SV34** 5’CCGCTCGGAGGAGAGCTCCCAGCCCATGGTC3’  **SV35** 5’GACCGGCTGAAAAGCCTGATC3’ | firefly-mCherry plasmid | CHIKV-Cbn *Sac*I *BamH*I | Replacement of DR(1+2) copies by a non-related 60%GC sequence. |
| Recombinant variants | Cloning between unique *Sac*I and *Not*I restriction sites in CHIKV-Cbn | **116** 5’CTAATCGTGGTGCTATGC3’  **122** 5’TTAGCGGCCGCTTTTTTTTTTTTTTTTTTTTTTTTTGAAATAT3’ |  |  | Introduction of the 3’UTR of selected variants into CHIKV-Cbn |
